# *Ixodes ricinus* as potential vector for Usutu virus

**DOI:** 10.1371/journal.pntd.0012172

**Published:** 2024-07-10

**Authors:** Julian W. Bakker, Emmanuelle Münger, Helen J. Esser, Reina S. Sikkema, Willem F. de Boer, Hein Sprong, Chantal B. E. M. Reusken, Ankje de Vries, Robert Kohl, Anne van der Linden, Arjan Stroo, Henk van der Jeugd, Gorben P. Pijlman, Marion P. G. Koopmans, Bas B. Oude Munnink, Constantianus J. M. Koenraadt

**Affiliations:** 1 Laboratory of Entomology, Wageningen University & Research, Wageningen, the Netherlands; 2 Department of Viroscience, Erasmus MC, Rotterdam, the Netherlands; 3 Wildlife Ecology and Conservation Group, Wageningen University & Research, Wageningen, the Netherlands; 4 Vogeltrekstation, Dutch Centre for Avian Migration and Demography, NIOO-KNAW, Wageningen, the Netherlands; 5 National Institute of Public Health and the Environment (RIVM), Utrecht, the Netherlands; 6 Centre for Monitoring of Vectors, Netherlands Food and Consumer Product Safety Authority (NVWA), Wageningen, the Netherlands; 7 Laboratory of Virology, Wageningen University & Research, Wageningen, the Netherlands; Faculty of Science, Ain Shams University (ASU), EGYPT

## Abstract

Usutu virus (USUV) is an emerging flavivirus that is maintained in an enzootic cycle with mosquitoes as vectors and birds as amplifying hosts. In Europe, the virus has caused mass mortality of wild birds, mainly among Common Blackbird (*Turdus merula*) populations. While mosquitoes are the primary vectors for USUV, Common Blackbirds and other avian species are exposed to other arthropod ectoparasites, such as ticks. It is unknown, however, if ticks can maintain and transmit USUV. We addressed this question using *in vitro* and *in vivo* experiments and field collected data. USUV replicated in IRE/CTVM19 *Ixodes ricinus* tick cells and in injected ticks. Moreover, *I*. *ricinus* nymphs acquired the virus via artificial membrane blood-feeding and maintained the virus for at least 70 days. Transstadial transmission of USUV from nymphs to adults was confirmed in 4.9% of the ticks. USUV disseminated from the midgut to the haemocoel, and was transmitted via the saliva of the tick during artificial membrane blood-feeding. We further explored the role of ticks by monitoring USUV in questing ticks and in ticks feeding on wild birds in the Netherlands between 2016 and 2019. In total, 622 wild birds and the *Ixodes* ticks they carried were tested for USUV RNA. Of these birds, 48 (7.7%) carried USUV-positive ticks. The presence of negative-sense USUV RNA in ticks, as confirmed via small RNA-sequencing, showed active virus replication. In contrast, we did not detect USUV in 15,381 questing ticks collected in 2017 and 2019. We conclude that *I*. *ricinus* can be infected with USUV and can transstadially and horizontally transmit USUV. However, in comparison to mosquito-borne transmission, the role of *I*. *ricinus* ticks in the epidemiology of USUV is expected to be minor.

## Author summary

Usutu virus (USUV) is a virus primarily circulating between birds and mosquitoes. Other blood-feeding arthropods, such as ticks, feed on birds. However, it is unknown whether ticks can be infected with USUV and transmit the virus to birds or other animals. Therefore, we infected a tick cell line and ticks collected from the field with USUV. We showed that the virus replicates in tick cells, as well as in field-collected ticks. Moreover, we showed that USUV disseminated through the tick body and was excreted with the saliva of ticks during artificial feeding. We further investigated if ticks are infected with USUV while feeding on wild birds and when searching for a host in the vegetation. We found that 7.7% of tick-infested birds carried USUV positive ticks, while USUV was not detected in questing ticks. Our findings provide evidence that ticks can be infected with USUV and might transmit the virus to another host, but the role of ticks in the transmission of USUV is expected to be minor.

## Introduction

Usutu virus (USUV) is a mosquito-borne flavivirus that has been spreading rapidly throughout Europe. USUV is known to have circulated in Europe since 1996 [1] and to date, USUV has been detected in a wide range of European countries [2–6]. The virus is maintained in a sylvatic cycle in which mosquitoes transmit the virus predominantly among birds. Wild bird species are the most important amplifying hosts of USUV and infection in birds can result in mortality [7]. The Common Blackbird (*Turdus merula*) is especially vulnerable to infection, and mass mortality of avian species has been reported during USUV outbreaks in multiple European countries [8–10]. In contrast to birds, humans and other mammals are thought to be dead-end hosts for USUV. Infection in humans is often asymptomatic and rarely results in clinical symptoms. Nevertheless, encephalitic and meningoencephalitic cases associated with USUV infection have been reported in Europe (reviewed in [11]). These cases occurred predominantly in immunocompromised patients or patients with underlying chronic diseases.

The primary arthropod vectors for USUV are ornithophilic *Culex* mosquitoes [7,12,13]. Besides mosquitoes, birds are exposed to other blood-feeding arthropods such as ticks [14]. In Europe, *Ixodes* ticks (principally *Ixodes ricinus*) are major vectors of tick-borne flaviviruses such as tick-borne encephalitis virus (TBEV). *Ixodes ricinus* is a generalist species feeding on a broad range of vertebrates [15]. Each active life stage (larva, nymph and female adult) needs a blood meal for their molt or, as adults, for the development of eggs. Larvae and nymphs generally feed on birds and on small mammals such as rodents, while adults feed on larger mammals such as deer [16]. Uninfected ticks can become infected when feeding on a viremic host (i.e. systemic transmission), or when feeding simultaneously with an infected tick on the same host, even if the host is not infected (i.e. non-systemic or cofeeding transmission) [15]. Transstadial transmission occurs when the virus is maintained in ticks molting from one life stage to the following life stage and is an important prerequisite for virus transmission to another vertebrate host following a subsequent bloodmeal.

Studies on the potential role of ticks as vectors of mosquito-borne flaviviruses have focused on the vector competence of ticks for West Nile virus (WNV) and St. Louis encephalitis virus (SLEV). WNV, SLEV and USUV are closely related viruses within the Japanese encephalitis serogroup and have similar transmission cycles. Laboratory studies showed replication of SLEV in *Amblyomma* ticks and WNV replication in *Amblyomma*, *Ixodes* and *Dermacentor* ticks [17–20]. In addition, WNV has been detected in *Ixodes* ticks that were either questing or feeding on rodents [21,22], and in *Amblyomma*, *Hyalomma*, *Ixodes* and *Rhipicephalus* ticks collected from birds and livestock [21,23,24]. Despite these findings, the exact role of ticks in the transmission of WNV and SLEV is not understood.

Identifying all vectors and host species that play a role in the transmission of zoonotic viral pathogens is essential to understand the dynamics of diseases and to guide surveillance and control measures. However, it is not known whether ticks are involved in the transmission of USUV. USUV has, thus far, not been detected in ticks collected from wild birds [25,26]. Nevertheless, as other mosquito-borne flaviviruses have been isolated from ticks, the question remains if USUV can infect ticks and if ticks can play a role in the transmission of USUV. To address this question, we experimentally exposed *I*. *ricinus* tick cells and field-collected ticks to USUV to assess the vector competence of these ticks. Furthermore, 622 tick-infecting birds and over 15,000 questing ticks were collected from multiple habitats in the Netherlands between 2016 and 2019 and screened for the presence of USUV.

## Materials and methods

### Ethics statement

All animal experiments were approved by the Dutch Central Authority for Scientific Procedures on Animals. The procedures for obtaining cow blood were approved by the Animal Ethics Committees of Wageningen Research (Permit Number: AVD1040020173624). The procedures for bird sampling were approved by the Animal Welfare Committees of the Royal Netherlands Academy of Arts and Sciences (Permit Number: AVD801002015342).

### Cell lines and viruses

*Ixodes ricinus* IRE/CTVM19 cells (Tick Cell Biobank, Liverpool, UK) were cultured at 28 °C in Leibovitz L-15 medium (Gibco) supplemented with 20% heat-inactivated fetal bovine serum (FBS, Gibco), 10% Tryptose phosphate broth (TPB), 2mM L-glutamine, 100 U/mL penicillin and 100 μg/mL streptomycin [27]. African green monkey kidney Vero E6 cells (ATCC CRL-1586) and baby hamster kidney BHK-21J cells were cultured in Hepes buffered Dulbecco’s modified Eagle medium (DMEM-Hepes, Gibco) supplemented with 10% FBS, 100 U/mL penicillin and 100 μg/mL streptomycin. Cells were maintained at 37 °C and 5% CO_2_. Vero E6 cells infected with tick homogenates were supplemented with DMEM-Hepes medium with gentamycin (50μg/mL) and Fungizone (2.5 μg/mL of amphotericin B and 2.1 μg/mL of sodium deoxycholate, Gibco). This medium will hereafter be called ‘DMEM complete’.

A passage 6 (P6) stock of USUV NL (Lineage 3 Africa, GenBank accession no. MH891847), a P3 stock of USUV Italy (Bologna, 2009, Lineage 2 Europe, GenBank accession no. HM569263, a P2 stock of WNV Greece (Lineage 2, GenBank accession no. HQ537483) and a P3 stock of tick-borne encephalitis virus TBEV Neudoerfl (kindly provided by the National Institute of Public Health and the Environment (RIVM), GenBank accession no. U27495) were used for infection experiments. All virus stocks were grown on BHK-21J cells and viral titres were determined using end-point dilution assays (EDPA) on Vero E6 cells.

### Virus growth curve analysis

To assess the replication of USUV NL and TBEV Neudoerfl in mammalian cells, Vero E6 cells were seeded at a confluency of 60–70% in six-wells plates one day before infection and infected at a multiplicity of infection (MOI) of 0.1. Supernatants were harvested at 0, 1, 2, 3 and 4 days post infection (dpi) to determine viral titres by EPDA.

To assess the replication of USUV Italy, USUV NL, WNV Greece and TBEV Neudoerfl in tick cells, IRE/CTVM19 *I*. *ricinus* cells were infected at a MOI of 0.1. Briefly, IRE/CTVM19 cells were seeded to a density of 5 x 10^5^ cells/mL in a total volume of 2.2 mL in flat-sided cell culture tubes (Nunc) and incubated with WNV (P3), USUV Italy (P2) or USUV NL (P6). One hour after incubation, cells were washed with PBS by centrifugation at 1,000 *g* for 5 min. Cells were resuspended in cell culture medium and aliquots of 50 μl were taken at day 0 (directly after medium replacement), 1, 2, 4, 7, 10 and 14 dpi to determine viral titres by EPDA.

### Ticks used for experimental infection with USUV

*Ixodes ricinus* nymphs for experiments with blood-feeding and artificial injection of USUV were collected by blanket-dragging using a 1 m^2^ white cloth. Ticks were collected between September 2019 and August 2020 in a mixed forest in the Dorschkamp, Wageningen, the Netherlands (51°58’38.9”N 5°41’58.4”E). Ticks were stored in an incubator at 18 °C with 99% relative humidity (RH) and 16:8 light:dark cycle in batches per 25 nymphs in 15 mL tubes (Falcon) with pierced lids. Ticks were stored for one to three weeks before use in either the artificial injection or blood-feeding assay.

### USUV infection via rectal pore injection

To study the replication and transmission of USUV in *I*. *ricinus*, nymphs and adult ticks were injected with 69 nL or 128nL, respectively, of a P6 stock of USUV NL (3.56 x 10^7^ TCID50/mL) using a Drummond Nanoject II Auto Nanoliter Injector (Drummond Scientific Company, USA). Glass capillaries (3.5” Drummond # 3-000-203-G/C, Drummond Scientific Company, USA) were prepared using a Narishige needle puller (model PB-7, Narishige, Japan). Ticks were immobilized on double-sided tape with the ventral side up. The fine point capillary was carefully inserted into the rectal pore of the tick and injected with virus stock. Ticks were removed from the tape using blunt-end tweezers. A subset of ticks was sacrificed by storage at -80 °C immediately after injection (0 dpi) in order to verify the presence of virus after injection. The other ticks were placed per five ticks in a 1.5 mL Eppendorf tube with pierced lid in an incubator at 21 ± 1 °C, 99% RH, and a 16:8 light:dark cycle. Ticks were removed from the incubator and sacrificed 14 days post injection. Because of sufficient numbers of individuals, nymphs were sacrificed at an additional timepoint, 28 days post injection. For adult ticks, only one time point after injection (14 days) was included.For all injected ticks, the presence of infectious virus was first determined by viral infectivity assays with mock-injected ticks used as control. For infected ticks, viral titres were determined using EPDAs.

### Artificial membrane blood-feeding system

An artificial membrane blood-feeding system was adapted from Oliver et al. [28] and Krull et al. [29] with several modifications as described in [30]. The blood-feeding unit consisted of a polycarbonate tube (50 x 30 x 2 mm, Flexinplex kunstoffen, the Netherlands) glued to a silicon membrane and closed with a cultivation plug for *Drosophila*. The blood-feeding unit was glued to a lid that could be screwed onto the container of the blood meal. The silicon membrane was made by using lens-cleaning paper (120 x 70 mm, Tiffen Lens Cleaning Tissue). The lens-cleaning paper was fixed to a transparent acetate A4 sheet on a flat surface and a silicon rubber was spread onto the cleaning paper. Briefly, a 10 mL mixture of components A and B of Ecoflex 00–10 soft rubber (Smooth-On, Inc.) were mixed and supplemented with 2 mL n-hexane (Sigma Aldrich). The mixture was spread over the lens cleaning paper by using a thin putty knife and the excess silicon rubber was scraped off resulting in a 50–70 μm thick membrane. Membranes were allowed to dry for a minimum of 12 h after which the polycarbonate tubes were glued on the membranes using ELASTOSIL E4 silicone glue (Wacker, Munich, Germany). The silicone glue was dried overnight, and membrane integrity was tested by adding 5-10mL of 70% ethanol to the assembled feeding units for 15 min after which the membranes were checked for leakage. In contrast with previous studies using artificial membrane blood-feeding units, no tick frass or physical stimuli were used [28,29,31]. For blood-feeding, 75–125 nymphs were added to each feeding unit.

### USUV artificial membrane blood-feeding

Sterile, heparinized cow blood was used for the artificial membrane blood-feeding experiments. Cow blood was obtained from Carus (Wageningen University, the Netherlands) under animal ethics protocol no. AVD1040020173624. Briefly, 3.5 mL of blood was supplemented with 4 mg/mL glucose solution to stabilize blood cells, gentamycin (50ug/mL) and Fungizone (2.5 μg/mL of amphotericin B and 2.1 μg/mL of sodium deoxycholate, Gibco). A P6 stock of USUV NL strain was diluted in 500 μL DMEM medium to a titre of 2 x 10^7^ TCID_50_/mL and added to 3.5 mL blood (final titre of 5 x 10^6^ TCID_50_ /mL blood). Virus spiked blood was replaced every 24 h. Before the blood change, membranes were rinsed by using a 0.9% NaCl solution. Ticks were allowed to feed for a maximum of nine days. Detached ticks were removed from the feeding unit by blunt-end tweezers and individually placed in 1.5 mL Eppendorf tubes with a pierced lid. The tubes were stored in a container with 99% RH, 21 °C and 16:8 light:dark cycle for 14, 28 or 70 days post engorgement (dpe). Ticks were stored in a -80 °C freezer after their incubation period. A subset of ticks was immediately stored after they were fully engorged and detached from the artificial membranes (0 days post engorgement) to confirm whether they ingested virus with the blood meal. Most of the ticks (90%) at 70 dpe molted into either male or female adult ticks.

### Dissemination of USUV in *Ixodes ricinus* nymphs

*Ixodes ricinus* nymphs were infected with USUV via artificial membrane blood feeding as described previously. Nymphs were stored for 7 to 10 days at 99% RH, 21 ± 1 °C and 16:8 light:dark cycle, after which engorged nymphs were collected and their legs were removed using a sterile scalpel. Legs and bodies were stored separately at -80 °C before viral infectivity assay.

### Transmission of USUV in a blood-meal

Nymphs were rectally injected with a P6 stock of USUV NL to study the transmission of USUV to a blood-meal. Nymphs were incubated for 14 days after which they were placed onto an artificial membrane blood-feeding unit. Thirty to 40 ticks per feeding unit were allowed to feed for six days. Similar methods were used as previously described with the exception that only 3 mL of blood was used instead of 4 mL and the blood was not spiked with virus. Blood was changed every 24 h and to 5 days of feeding, 500μl of blood was collected and mixed with Trizol LS for RNA isolation. After RNA isolation, a real-time reverse transcription PCR (RT-qPCR) for USUV [32] was used for the detection of USUV in the blood.

### Infectivity assay

Frozen complete ticks, ticks with legs cut off, or tick legs only were homogenized using a combination of zirconium oxide beads (2.0 mm) and stainless steel beads (0.9–2.0 mm) in a Bullet blender (Next Advance, USA). Briefly, samples were homogenized for 2 min, spun down for 1 min at 12,000 *g* in an Eppendorf 4125 centrifuge. Next, 100 μL of DMEM-HEPES complete medium was added and samples were again homogenized for 2 min at max speed and spun down for 1.5 min at 12,000 *g*. For each tick homogenate, 30 μL was added to a Vero E6 monolayer of 70–80% confluency in a 96-wells plate. After 2 hours, the medium was removed, washed once with 1x PBS, and replaced with 100 μL DMEM-HEPES complete medium. Cytopathic effect (CPE) was scored 6 days post infection (dpi).

### End point dilution assay (EPDA)

Viral titres in TCID_50_/mL were determined using 10μL of supernatant or tick homogenate in an EPDA. Briefly, Vero E6 cells were detached using Trypsin (Gibco) and diluted to 5 x 10^5^ cells/mL in DMEM-HEPES complete or DMEM-HEPES standard cell culture medium (for cell supernatant). Virus samples were tenfold serially diluted (10^−1^ up to 10^−8^) in DMEM-HEPES complete. The Vero E6 cells were added in a 1:1 ratio to the virus dilutions and 10 μL of each virus suspension was added to six wells of a 60-well MicroWell Plate (Nunc, Roskilde, Denmark). CPE was scored at 6 dpi and the TCID_50_/mL was calculated according to Reed and Muench [33]. The value of 1 x 10^3^ TCID_50_/mL, which corresponds to the detection limit of the EPDA, was assigned as viral titre for ticks determined USUV positive by the infectivity assay but with a viral titre below the detection limit of the EPDA.

### Deep sequencing of small RNAs

Total RNA was extracted from three pools of *Ixodes* ticks. One pool of ticks consisted of two female and one male tick (pool 1), a second pool of ticks consisted of four female ticks (pool 2); these two pools consisted of USUV-positive ticks from the USUV artificial membrane blood-feeding experiment at 70 dpe. A third pool consisted of four USUV RNA positive tick pools (22 ticks in total) from four wild birds. Total RNA of pool 1 and pool 2 was isolated from the supernatant of homogenized ticks using Trizol LS. Total RNA of pool 3 was isolated using the ALL PREP RNA mini kit (Qiagen). Small RNA libraries were generated from 20 μl of tick total RNA with a concentration of 110 ng/μl for pool 1 and 2 and 220 ng/μl for pool 3 on a DNBSeq UMI platform from BGI (Shenzhen, Guangdong, Hongkong). Single-end FASTQ reads were generated using an in-house filtering protocol of BGI.

### Small RNA analyses

Small RNA sequencing library analysis was performed as previously described by Abbo et al. [34] using the Galaxy webserver [35]. Small RNA reads were mapped using Bowtie 2 version 2.3.4.1 allowing 1 mismatch and a seed length of 28. Reads were mapped to the viral genomes of USUV NL (Accession no. MH891847) with a 3’ UTR sequence from an USUV isolate from Italy (Accession no. KX816650) and 5’ UTR sequence from a closely related USUV isolated in the Netherlands (Accession no. KY128482). Size distribution profiles of mapped virus-derived small interfering RNA (vsiRNA) reads were made. Next, the 5’ ends of the 22-nt vsiRNA were mapped to the USUV genome. Virus-derived Piwi-interacting RNAs (piRNAs) of 24–30 nt were mapped to the USUV genome. Read counts for the size distribution profiles and the genome distributions were normalized against the total reads per library (21 million).

### Sampling of wild birds and feeding ticks

As part of the surveillance system for zoonotic viruses in birds of the Netherlands [4,36], live wild birds were captured for ringing and sample collection between March 2016 and December 2019, at different locations throughout the country. A throat swab and, for larger birds, also a cloacal swab were collected. Throat and cloacal swabs were pooled and stored in virus transport medium. Birds were examined for tick infestation and ticks were removed using tweezers. Ticks were stored alive in 2 mL tubes. Bird swabs and ticks were shipped to the laboratory at ambient temperature in a polyvinylchloride envelope through a medical postal service. Ticks were frozen at -80°C upon arrival until further processing. Sampling was performed under ethical permit AVD801002015342 issued to NIOO-KNAW.

### Nucleic acid extraction and USUV diagnostics from tick and bird samples

Complete tick bodies were processed individually or in pools of up to seven individuals infesting a unique bird. For a selection of ticks (n = 35), legs of the ticks were removed using a sterile scalpel, and legs and bodies were processed separately. Genomic DNA and total RNA were isolated from ticks using the AllPrep DNA/RNA Micro kit (Qiagen) according to the manufacturer’s instructions. In short, 350 μl lysis buffer (1% Beta-mercaptoethanol -Merck Millipore- in Buffer RLT) was added to the ticks in 2 ml Lysing Matrix H tubes (MP Biomedicals), after which they were disrupted using the FastPrep bead beater (MP Biomedicals) shaking at 6.5 m/s for 2 times 60 seconds. Homogenates were cleared by centrifugation and the supernatants were used for nucleic acid extraction.

Total NA was extracted from bird swabs, using the MagNA Pure 96 and DNA and Viral NA Large Volume Kit (Roche) according to the manufacturer’s instructions. RNA samples from birds and ticks were screened for the presence of USUV using RT-qPCRs described by Nikolay et al. [37] and Jöst et al. [2]. Phocine distemper virus was used as an internal control.

### Molecular identification of tick species from wild birds

Taxonomic assignment of ticks was performed on extracted genomic DNA, through PCR amplification and Sanger sequencing of the mitochondrial cytochrome c oxidase subunit one (CO1) gene [38]. Amplification reactions were performed using the HotStarTaq DNA Polymerase and Buffer (Qiagen). The following thermal cycling program was used: 95°C for 15 min, 35 cycles at 95°C for 1 min, 48°C for 1 min, and 72°C for 1 min, followed by a final extension at 72°C for 10 min. PCR products were visualized by agarose gel electrophoresis. PCR products were purified and sequenced using Sanger sequencing technology. The *CO1* sequences were analyzed using BLAST [39] for species identification.

### USUV whole genome sequencing and sequence data analysis

USUV genomes were generated using a multiplex PCR for Oxford Nanopore sequencing as previously described [36,40]. In short, random primers (Invitrogen) were used to perform reverse transcription using ProtoScript II (NEB, cat. No. E6569) after which USUV specific multiplex PCR was performed in two reactions using Q5 Hot Start High-Fidelity DNA Polymerase (NEB, cat no. M0493). Nanopore sequencing was performed according to manufacturer’s instructions using the 1D Native barcoding genomic DNA Kit (Nanopore, EXP-NBD103 and SQK-LSK108) on a FLO-MIN106 flow cell. A total of 12 or 24 samples were multiplexed per sequence run. Seventeen tick samples positive for USUV RNA (eight single ticks and nine pools) with CT values ranging between 19 and 30 were subjected to USUV Multiplex PCR and, if amplification was successful, to sequencing. Raw sequence data were demultiplexed using Porechop [41]. Reads were quality controlled to a minimum length of 150 and a median PHRED score of 10 using FastP [42]. A reference-based alignment was performed using Minimap2 [43]. A consensus genome was extracted, reads were remapped to this consensus sequence, and a new consensus sequence was generated. Positions with <100 coverage were replaced with an ‘N’. Homopolymeric and primer binding regions were manually checked and resolved by consulting reference genomes. Positions with major variant assigned but representing ≤ 70% of the coverage depth were manually checked and replaced by an ambiguous nucleotide if a truly ambiguous position. The USUV genome sequences from this study have been deposited in the GenBank [44] database under the accession numbers OP921076 to OP921083 (S2 Table). For one tick sample where Oxford Nanopore Technologies MinION sequencing resulted in sequence with coverage gaps, one gap was closed using Sanger sequencing as described under the tick species identification section.

All available full-length USUV genomes were retrieved from GenBank [44] and aligned with the newly obtained USUV sequences using MUSCLE [45]. IQ-TREE was used to perform maximum likelihood phylogenetic analysis under the GTR + I + G4 model as best predicted model using the ultrafast bootstrap option with 1,000 replicates [46].

### Surveillance of USUV in questing ticks

Questing ticks were collected in 2017 and 2019 by blanket dragging. Ticks were collected from 29 sampling locations in 2017 and 2019. Locations were selected based on known [47] and potential [48] TBEV transmission sites (S1 Table). Ticks were processed in pools consisting of either 25 nymphs, four individual adult female ticks, or eight adult male ticks. After homogenization of the tick pools using Lysis matrix Z (MPbiomedicals) and the Fast prep FP120 homogenizer (Thermo Savant, Carlsbad, USA), RNA was extracted using the automated MagNA Pure 96 system (Roche). RNA samples from ticks were analyzed for USUV using RT-qPCR. Forward primer USUV-F2 (GACATCGTTCTCGACTTTGACTATTA), reverse primer USUV-R2 (GCTAGTAGTAGTTCTTATGGAGGGT) and USUV probe USUV-P2 (CACCGTCACAATCACTGAAGCATGTG) were used together with the TaqMan Fast Virus 1-Step Master Mix (Thermo Fisher).

### Statistical analyses

Generalized linear mixed models (GLMMs) with a truncated negative binomial distribution and log-link function were used to test the effect of incubation period on USUV titres. Overdispersion of viral titres was addressed by including individual ticks as random factor. Incubation period and replicate of the experiments were included as fixed factors. Generalized linear models (GLMs) with a binomial distribution and logit link function were used to test for the effect of incubation period on USUV infection. Incubation period and replicates of the experiment were included as fixed factors. The effect of dissemination on USUV infection was tested using a GLM with binomial distribution and logit link function. Incubation period, infection in tick body versus legs, and replicate were included as fixed factors. Model diagnostics were performed using the “DHARMa” package [49]. GLMs were constructed using the R package “glmmTMB” [50]. Estimated marginal mean viral titres and estimated probabilities of infection rates were calculated using the package “emmeans”[51]. Regression coefficients and intervals were back-transformed to obtain Incidence Rate Ratios (IRR) for differences in viral titres and Odds Ratios (OR) for differences in infection probability. Pairwise contrasts of significant effects were performed with a Tukey HSD adjustment for multiple comparisons. All statistical analyses were carried out with the statistical software package R version 4.2.0 [52] using the R studio platform [53].

## Results

### Growth kinetics for mosquito- and tick-borne viruses in mammalian and tick cell lines

**Fig 1:**
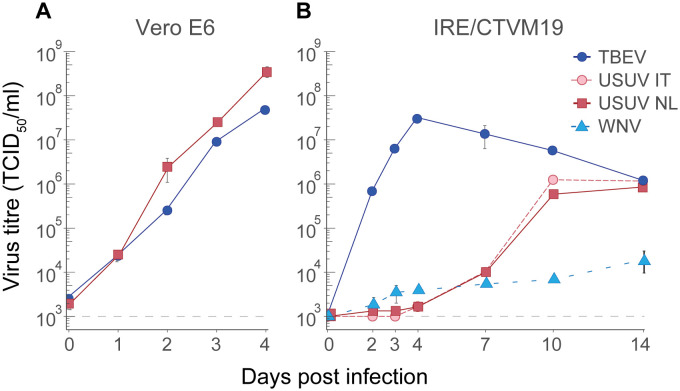
Growth kinetics of tick- and mosquito-borne viruses in mammalian Vero E6 and *Ixodes ricinus* IRE/CTVM19 tick cell lines. Vero E6 (A) and IRE/CTVM19 (B) cells were infected with a multiplicity of infection (MOI) of 0.1 with Usutu virus (USUV), West-Nile virus (WNV) and tick-borne encephalitis virus (TBEV). Viral titres were determined using end-point dilution assays (EPDAs). The results are shown as the mean viral titres ± standard error of three replicates. Dashed line indicates the detection limit of the EPDA at 1 x 10^3^ TCID_50_/mL.

First, the *in vitro* growth kinetics of USUV were compared with those of the mosquito-borne WNV and the tick-borne TBEV. In Vero cells, USUV NL reached a titre of 3.56 x 10^8^ TCID_50_/mL and TBEV reached a titre of 3.56 x 10^7^ TCID_50_/mL, both at 4 days post-infection (dpi) (Fig 1A). However, when an *I*. *ricinus* IRE/CTVM19 cell line was infected with WNV, two strains of USUV (USUV NL and USUV Italy), and TBEV, a clear difference in growth kinetics was observed: TBEV reached a peak titre (3.2 x 10^7^ TCID_50_/mL) at 4 dpi, whereas USUV Italy and USUV NL reached a substantially lower peak titre at 14 dpi (8.6 x 10^5^ TCID_50_/mL and 1.1 x 10^5^ TCID_50_/mL, respectively, Fig 1B). WNV reached a titre of 1.1 x 10^4^ TCID_50_/mL at 14 dpi and replicated slower in IRE/CTVM19 tick cells compared to USUV.

### USUV artificial infection of *Ixodes ricinus* via injection

**Fig 2:**
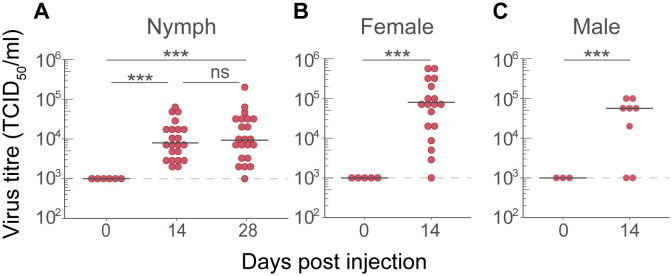
Viral titres of USUV injected *Ixodes ricinus* nymphs (A), females (B) and males (C). Ticks were injected with USUV. Groups of ticks were sacrificed at 0, 14 and 28 (only nymphs) days post injection. Viral titres were determined using end-point dilution assays (EPDAs). Each data point represents one injected tick. Median titres are shown as horizontal black lines. A dashed line indicates the detection limit of the EPDA at 1 x 10^3^ TCID_50_/mL. All samples were positive for USUV based on infectivity assay. Samples with a viral load below the detection limit of the EPDA are represented on the detection limit line. Indicated statistics show the output of GLMMs (ns = not significant, *** p < 0.001).

To further assess the replication of USUV *in vivo*, nymphal and adult *I*. *ricinus* ticks were injected with USUV-NL. Nymphal and adult ticks received an initial viral dose of 2.4 x 10^3^ TCID_50_ or 4.8 x 10^3^ TCID_50_ respectively. At day 0 (immediately after injection), ticks were determined positive for USUV by infectivity assay, however, the estimated marginal mean viral titres were below the limit of detection of the EPDA (1 x 10^3^ TCID_50_/mL, Fig 2A). The estimated marginal mean viral titres significantly increased from 1 x 10^3^ TCID_50_/mL at 0 days post injection to 1.5 x 10^4^ TCID_50_/mL at 14 days post injection (IRR, 5.79, 95% CI: 4.81–46.00, p < 0.001, Fig 2A). Estimated marginal mean viral titres increased further to 2.5 x 10^4^ TCID_50_/mL at 28 dpi (IRR, 6.78, 95% CI: 7.68–74.01, p < 0.001), but the difference between 14 dpi and 28 days post injection was not significant (IRR, 1.54, 95% CI: 0.763–3.36, p = 0.28). In addition, estimated marginal mean viral titres significantly increased between 0 and 14 days post injection in both female and male adult ticks (from 1 x 10^3^ TCID_50_/mL to 1.4 x 10^5^ TCID_50_/mL, LRT for females, χ^2^ = 20.16, df = 1, p < 0.01 and to 4.1 x 10^4^ TCID_50_/mL for males, LRT, χ^2^ = 10.18, df = 1, p < 0.01, Fig 2B and 2C). These results indicate that USUV actively infected the ticks.

### USUV artificial infection of *Ixodes ricinus* via bloodmeal

**Fig 3:**
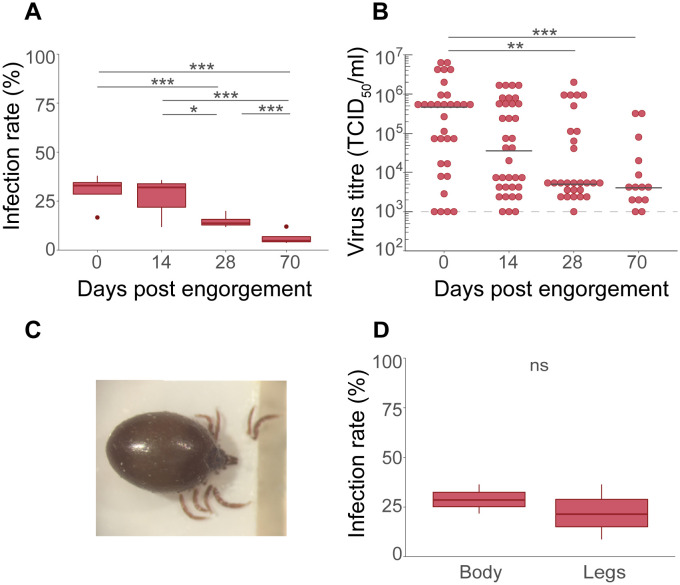
Artificial infection of *Ixodes ricinus* with USUV via a bloodmeal. (**A**) Infection rates of USUV blood-fed nymphs at 0 (n = 152), 14 (n = 112), 28 (n = 182) and 70 (n = 318) days post engorgement. Boxplots represent four independent replicates. (**B**) Viral titres of USUV infected ticks at 0, 14, 28 and 70 dpe, determined using end-point dilution assays (EPDAs). Each dot represents a single tick. Median titres are shown as horizontal black lines. All samples were positive for USUV based on infectivity assay. Samples with a viral load below detection limit of the EPDA are represented on the detection limit line. (**C**) Engorged nymphs with legs removed. (**D**) Infection rate of USUV in body and legs of *I*. *ricinus* nymphs (n = 124) after 7–10 days post USUV infected blood feeding. Boxplots represent three independent experiments. Indicated statistics show the output of GLMMs. (ns = not significant, * p < 0.05, ** p < 0.01, *** p < 0.001). Dashed lines indicate the detection limit of the end-point dilution assay at 1 x 10^3^ TCID_50_/mL.

Next, *I*. *ricinus* nymphs were infected using an artificial membrane blood-feeding system to determine if *I*. *ricinus* can acquire USUV via the oral route, and if USUV can persist transstadially in ticks. USUV infection status was followed over a period of 70 days post-engorgement (dpe). At 70 dpe, 89.9% (286/318) of the nymphal ticks had molted into adults. The time after engorgement (incubation period) had a significant negative effect on infection rates (LRT, χ^2^ = 84.49, df = 3, p < 0.001, Fig 3A). At 0 dpe, 35.1% (95% CI: 27.9–43.2) of the ticks were infected with USUV and this decreased to 31.3% (95% CI: 23.3–40.4; OR = 1.16, 95% CI: 0.59–2.38, p = 0.91) at 14 dpe, to 15.4% (95% CI: 10.8–21.4; OR = 2.80, 95% CI: 1.38–5.68, p < 0.001) at 28 dpe and to 5.0% (95% CI: 3.1–8.1; OR = 3.51, 95% CI: 1.49–8.23, p < 0.001) at 70 dpe (Fig 3A). While controlling for a significant effect of experimental replicates (LRT, *χ*^2^ = 8.75, df = 3, p < 0.05), viral titres significantly decreased over time in ticks where USUV could still be detected (LRT, *χ*^2^ = 20.25, df = 3, p < 0.001, Fig 3B). USUV titres were similar at 0 and 14 days of incubation but significantly dropped after 28 and after transstadial transmission at 70 days of incubation (Tables 1 and S3). Transstadial transmission of USUV was detected in 4.9% (14/286) of the adult ticks.

**Table 1:** Estimated marginal mean USUV titres in *Ixodes ricinus* blood-fed ticks.

Incubation period (days)	Estimated marginal mean titre	95% CI
0	1.1 x 10^6^ TCID_50_/mL	5.1 x 10^5^–2.7 x 10^6^ TCID_50_/mL
14	5.0 x 10^5^ TCID_50_/mL	2.0 x 10^5^–1.2 x 10^6^ TCID_50_/mL
28	1.24 x 10^5^ TCID_50_/mL	5.0 x 10^4^–3.1 x 10^5^ TCID_50_/mL
70	6.5 x 10^4^ TCID_50_/mL	2.2 x 10^4^–1.9 x 10^5^ TCID_50_/mL

### USUV dissemination by *Ixodes ricinus*

*Ixodes ricinus* nymphs were infected with USUV using an infectious bloodmeal to test if USUV can overcome the midgut-barrier and disseminate into the haemocoel. After 7 to 10 dpe, 27.4% (17/62) of the ticks had USUV-infected bodies and 19.3% (12/62) had both infected legs and bodies (Fig 3C and 3D). Not every tick infected with USUV in the body had USUV-positive legs, however, the infection rate in tick bodies was not significantly different from that in the tick legs (LRT, χ^2^ = 1.16, df = 1, p = 0.28). This indicates that USUV generally crossed the midgut-barrier and disseminated into the haemocoel.

### USUV transmission by *Ixodes ricinus*

To further test whether USUV infected ticks may transmit the virus via their saliva, ticks infected by injection were placed to feed on a blood-feeding unit, and blood from the unit was tested for the presence of USUV (Table 2). Only a limited number of ticks started feeding on the membrane, which could be caused by the physical damage of the tick after injection, but USUV RNA was detected in two out of six feeders indicating that ticks have the capacity to transmit the virus after being infected.

**Table 2:** Transmission of USUV RNA to blood in artificial feeding units.

Feeding unit #	N USUV injected ticks in feeder	N of feeding ticks	Ct values of USUV RNA*				
			Day 1	Day 2	Day 3	Day 4	Day 5
1	30	7	ND	ND	33.2	32.3	ND (35.2)
2	30	1	ND	ND	ND	ND	ND
3	40	1	ND	ND	ND	ND	ND
4	30	2	ND	ND	ND	ND	ND
5	35	7	ND	ND	ND	ND	ND
6	30	8	ND	ND	ND	32.6	33.8

*Ticks were placed on the feeders at day 0, however, not all ticks started feeding at this day. Therefore, we cannot conclude whether or not the likelihood of USUV RNA detection increases or decreases with the time of feeding. ND = no USUV RNA detected. A conservative cut-off values of Ct 34 was used as described by Wang et al. [59].

### Detection of USUV RNA in ticks feeding on birds and in questing ticks

**Fig 4:**
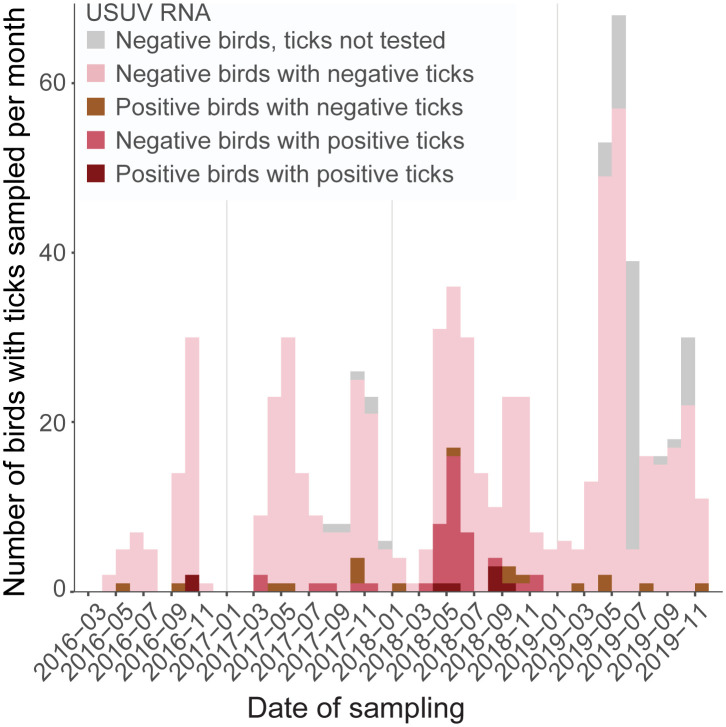
Time series of USUV RNA detections in tick-infested wild birds and carried ticks in the Netherlands, 2016–2019. The number of birds with ticks sampled and tested for the presence of USUV RNA is shown per month. Colors of the bars indicate the combinations of results in bird and carried ticks. One bird can carry multiple ticks.

The potential role of ticks in the transmission of USUV was investigated by screening ticks feeding on wild birds for the presence of USUV RNA. Between March 2016 and December 2019, 5,931 wild birds from 107 different species were captured and tested for USUV. A total of 687 birds (11.6%) from 24 different species and 13 families carried at least one tick (Table 3). Ticks were collected on birds all year long, with highest numbers between April and November (Fig 4). Of all the tick-infested birds, 273 (39.7%) carried a single tick, 151 (22.0%) carried two ticks and 263 (38.3%) carried more than two ticks. The largest number of ticks from a single bird (n = 35 ticks) was collected from a Common Blackbird. Two species from the Turdidae family were most often infested by ticks, Common Blackbirds (467/1794, 26.0%) and Song Thrushes (*Turdus philomelos*, 97/392, 24.7%). Infested birds carried *I*. *ricinus* ticks (323/403, 80.1%) or *Ixodes frontalis* (81/403, 20.1%) (Table 3). *Ixodes lividus* was found only twice, on sand martins (*Riparia riparia*).

**Table 3:** Wild bird species infested by ticks in the Netherlands, 2016–2019.

Bird family	N birds captured	N birds carrying ticks	Proportion of birds carrying ticks (%)	N Ticks per bird, median (range)*	N Birds with ticks identified	*I*. *ricinus* **	*I*. frontalis**	*I*. *ricinus* and *I*. frontalis**	*I*. *lividus* **
Turdidae	2248	575	25.6	2 (1–35)	348	283 (81.3%)	62 (17.8%)	3 (0.9%)	0
Sylviidae	1224	43	3.5	1 (1–14)	21	18 (85.7%)	3 (14.3%)	0	0
Acrocephalidae	591	33	5.6	1 (1–5)	12	11 (91.7%)	1 (8.3%)	0	0
Muscicapidae	182	10	5.5	1 (1–3)	5	3 (60.0%)	2 (40.0%)	0	0
Passeridae	480	8	1.7	1 (1)	8	1 (12.5%)	7 (87.5%)	0	0
Hirundinidae	71	5	7.0	1 (1–2)	2	0	0	0	2 (100%)
Fringillidae	24	4	16.7	1 (1–2)	3	0	3 (100%)	0	0
Corvidae	232	3	1.3	1 (1–13)	0				
Paridae	37	2	5.4	2.5 (1–4)	2	2 (100%)	0	0	0
Charadriidae	5	1	20.0	1 (1–1)	1	1 (100%)	0	0	0
Ciconiidae	1	1	100.0	3 (3–3)	1	1 (100%)	0	0	0
Rallidae	101	1	1.0	1 (1)	0				
Strigidae	6	1	16.7	1 (1)	0				
**Total**	5931†	**687**	**11.6**	**2 (1–35)**	**403**	**320 (79.4%)**	**78 (19.4%)**	**3 (0.7%)**	**2 (0.5%)**

* Birds without ticks are excluded.

** Numbers indicate number of birds with the ticks identified as said species.

^†^ Total across all bird families, including bird families never found with ticks.

Ticks from 622 birds carrying *Ixodes* ticks were tested for the presence of USUV RNA. Twenty-five birds carrying ticks tested positive for USUV (4.0%, Table 4). Eight of these birds carried at least one USUV-positive tick. In addition, 40 bird swabs tested negative for USUV RNA but carried USUV-positive ticks. Thus, 48 out of 622 birds (7.7%) carried USUV-positive ticks. For two USUV-positive birds, carrying one and eight ticks, respectively, ticks were processed individually, and their legs were separated from the body. For both birds, USUV RNA was detected in legs of the feeding ticks (sample IDs 16TV1039 and 16TV113, S2 Table). For 34 of the 48 birds carrying USUV-positive ticks, the ticks could be assigned to species based on sequencing of the mitochondrial cytochrome c oxidase subunit one (CO1) gene [38]. In 31 cases, USUV-positive feeding ticks were identified as *I*. *ricinus* (seven cases with single ticks, 24 cases with ticks tested in pools) and in three cases as *I*. *frontalis* (three cases with single ticks).

**Fig 5:**
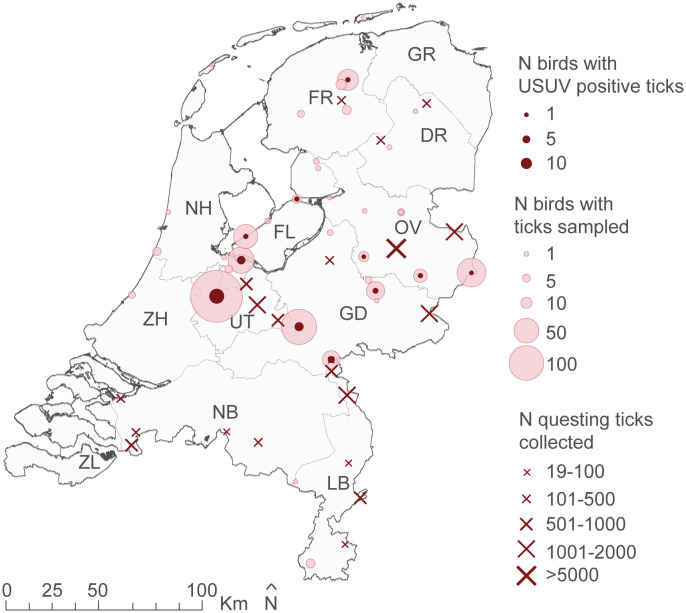
Geographical distribution of USUV RNA detection in ticks collected from wild birds and in questing ticks in the Netherlands, 2016–2019. Locations of sampling and of USUV RNA positive cases. Circles indicate sampling locations of birds carrying tick, size is proportional to the number of birds sampled. Crosses indicate collection locations of questing ticks, size is graduated by range of number of ticks collected. Provinces are labeled with two-letter abbreviation. Source administrative boundaries: CBS, Kadaster, "CBS Gebiedsindelingen 2019" (https://service.pdok.nl/cbs/gebiedsindelingen/atom/v1_0/index.xml). Created using the R packages “*sp*” and “*rgdal*” and ArcMap 10.8.1.

USUV RNA was detected in ticks collected from birds each year between 2016 and 2018 at multiple locations throughout the country (Figs 4 and 5). No USUV RNA-positive ticks were found in 2019. In spring 2018, there was a notable increase in the number of ticks collected that tested positive for USUV RNA (Fig 4), with a peak of 16 birds carrying USUV RNA-positive ticks in May 2018. In comparison, only two wild birds tested positive for USUV RNA in May 2018, one of which also carried USUV RNA-positive ticks. This increase in USUV RNA-positive ticks preceded the increase in reported dead blackbirds in the Netherlands, which started at the end of July [54].

In addition, we tested 15,381 questing ticks collected throughout the Netherlands between June and August 2017 and between April and June 2019 for USUV RNA (S1 Table and Fig 5). None of these ticks tested positive for USUV.

**Table 4:** USUV RNA detection in swabs collected from wild bird species infested by ticks, 2016–2019.

Bird species	Bird family	N positive birds with positive ticks	N negative birds with positive ticks	N positive birds with negative ticks	N negative birds with negative ticks	N negative birds, ticks not tested
Common Blackbird *Turdus merula*	Turdidae	6	34	14	383	30
Song Thrush *Turdus philomelos*	Turdidae	2	1	1	81	12
Common whitethroat *Sylvia communis*	Sylviidae	0	1	1	10	2
Eurasian Blackcap *Sylvia atricapilla*	Sylviidae	0	1	0	20	5
European Robin *Erithacus rubecula*	Muscicapidae	0	1	0	2	0
Icterine Warbler *Hippolais icterina*	Acrocephalidae	0	1	0	1	2
Marsh Warbler *Acrocephalus palustris*	Acrocephalidae	0	1	0	5	2
**Total**		**8**	**40**	**17**	**557** *	**65** *

*Total across all bird species carrying ticks, including species without RT-qPCR USUV positive results in birds or carried ticks

### Small RNA response to USUV infection in ticks

**Fig 6:**
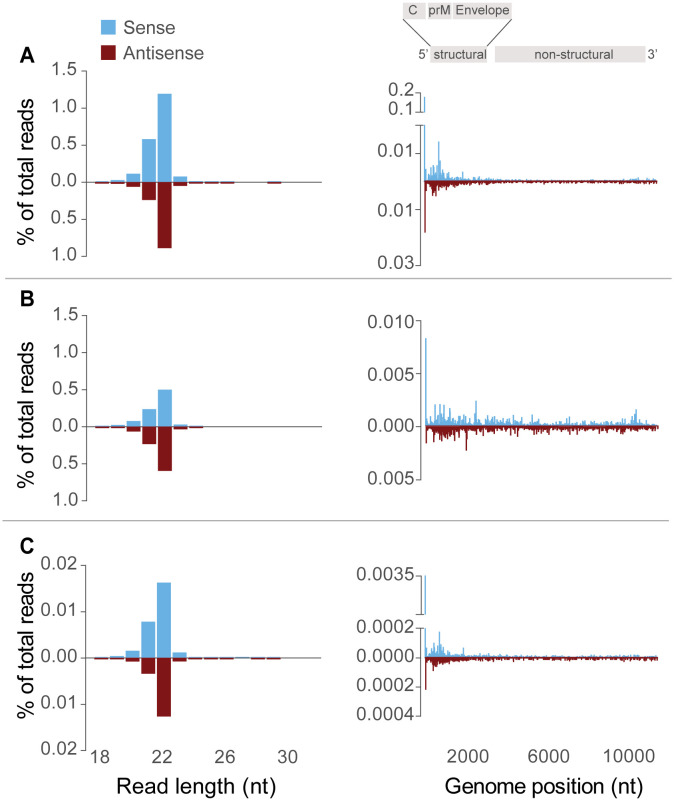
Size distribution of 18-32nt vsiRNAs and distribution of 22 nt vsiRNAs across the USUV genome. The percentages of reads mapping to the sense (blue) or antisense (red) genome are shown. Panels **A-C** show the size distribution of vsiRNAs mapping to USUV genome as well as the 22 nt vsiRNAs across the USUV genome. The 3’ and 5’ untranslated regions (UTRs) as well as the structural and non-structural regions of the USUV genome are depicted together with the Capsid (C), pre-Membrane (prM) and Envelope (E) protein coding regions. Panel **A** and **B** represent two pools of artificial membrane blood-fed *I*. *ricinus* ticks. Panel **C** represents a pool of USUV infected *Ixodes* ticks collected from wild birds. vsiRNA read counts were normalized against the total small RNA library (21 million reads).

The *I*. *ricinus* virus-derived small interfering RNA (vsiRNA) response was characterized in USUV-infected ticks. vsiRNAs are a product of the antiviral RNA interference pathway in arthropods [55]. Two tick pools experimentally infected with USUV and one tick pool with USUV RNA-positive ticks collected from wild birds were used. vsiRNA reads mapped to both sense and antisense genomes of USUV in all libraries (Fig 6). This indicates a strong antiviral response of *I*. *ricinus* against USUV. Furthermore, the presence of antisense vsiRNAs shows that USUV actively replicated in ticks. A peak of 22 nt vsiRNAs was observed in all pools. The 22 nt reads mapped at different sites along the USUV genome, but a large number of reads mapped to the region coding for USUV structural genes (Fig 6). All pools showed a 22 nt vsiRNA hotspot at the 5’UTR region. Peaks in the number of 22 nt reads mapped at specific regions of the genome (so-called vsiRNA ‘hotspots’) can likely be explained by the presence of hairpin structures [56]. No peak of 24–30 nt vsiRNAs, which is the size range of virus derived PIWI-interacting RNAs (piRNAs), was observed in any of the pools. The 24–30 nt vsiRNAs were distributed along the complete genome of USUV (S1 Fig).

### Phylogenetic analysis

Near complete or complete viral genomes were recovered from three *I*. *ricinus* infesting one Common Blackbird, one *I*. *frontalis* infesting one Common Blackbird and a pool of five ticks collected from one Song Thrush. All ticks from which USUV could be sequenced infested a bird that also tested USUV RNA positive. From these birds, USUV sequences were also generated (S2 Table). Attempts to obtain a viral genome from other USUV-positive ticks, including those collected from negative birds, were unsuccessful, which might be explained by low viral loads and prolonged sample storage.

**Fig 7:**
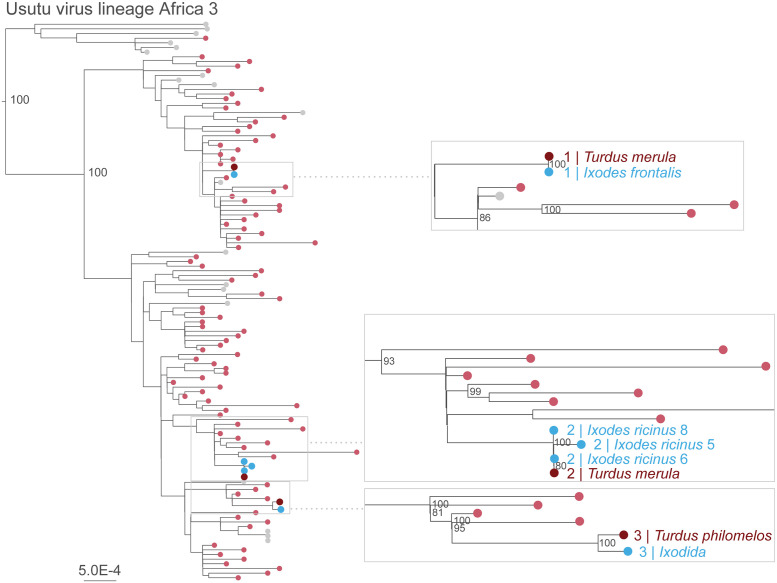
Phylogenetic analysis. Maximum likelihood phylogeny of USUV complete coding sequences, subset comprising sequences from lineage Africa 3. Sequences from the Netherlands are colored in light red. Panels show close-up views into regions of the maximum likelihood phylogenetic tree, highlighting the positions of sequences generated in this study; sequences derived from birds are shown in dark red and sequences derived from ticks carried by these birds are shown in blue, all are from the Netherlands. In the overview tree, only bootstraps of major internal nodes are indicated, in the zoom, bootstraps ≥80% of the external nodes are indicated. Scale units are nucleotide substitutions per site.

Phylogenetic analysis revealed that USUV detected in ticks and their bird host all belonged to lineage Africa 3 and were closely related to other USUV genome sequences from blackbirds in the Netherlands (Fig 7). The viral genome sequences obtained from the ticks clustered together with sequences obtained from their respective bird host; these were identical or very similar (maximum of two nucleotides difference between a sequence obtained from one tick and the sequence obtained from the bird host).

## Discussion

Identifying vectors that play a role in the transmission of an emerging virus is essential to understand the ecology of a disease. In the current study, the role of ticks in the transmission of USUV was evaluated using laboratory experiments and field-collected data. We showed that USUV can infect ticks *in vitro* and *in vivo*, that USUV was transmitted transstadially in 4.9% of infectious blood-fed *I*. *ricinus* ticks, and that USUV can be excreted with the saliva of ticks. Thereby, we demonstrate that USUV transmission from a tick to another host is possible. Furthermore, 7.7% of tick-infested birds sampled during a period of endemic USUV circulation carried USUV RNA positive ticks. However, in a field study collecting over 15,000 questing ticks, USUV RNA was not detected.

USUV injection in field-collected *I*. *ricinus* ticks resulted in virus replication in both nymphal and adult ticks. Furthermore, USUV blood-fed *I*. *ricinus* ticks remained infected with USUV after molting from nymphal to adult ticks, indicating transstadial transmission of the virus. Nevertheless, the percentage of transstadial transmission was low compared to that of WNV in adult *I*. *ricinus* ticks as observed in a recent study by Răileanu et al. [20] (4.9% for USUV versus 46.7% for WNV). In the current study we used infectivity assays and EPDAs on Vero cells for USUV detection. The combination of these methods has the advantage of directly quantifying infectious viral particles, but is less sensitive compared to USUV detection using RT-qPCR [18]. Our results may therefore be an under-estimation of the actual USUV infection in *I*. *ricinus* and may explain the low transstadial transmission rates when compared to the study of Răileanu et al., in which RT-qPCR was used for USUV detection [20]. Nevertheless, in a study by Anderson et al. [18], which used both RT-qPCR and Vero cell culture for the detection of WNV, a low fraction of transstadial transmission of WNV was found for *I*. *scapularis* larvae. An alternative explanation for the differences in transstadial transmission could be related to the origin of the ticks used. The study by Răileanu et al. [20] used laboratory-reared ticks whereas the study by Anderson et al. [18] and the current study used field-collected ticks. The large differences in transstadial transmission rates in these studies may be explained by the differences in physiological conditions of these ticks. Infection success of viruses in ticks after an infectious bloodmeal is influenced by tick origin and collection date [30,57]. Moreover, microbiota of arthropods differ between laboratory-reared and field-collected specimens [58]. The absence or presence of specific symbiotic bacteria in the midgut can influence the susceptibility of ticks for pathogens [59]. However, how the microbiota of ticks exactly influence the susceptibility of ticks to viruses is poorly understood.

During viral replication, double-stranded viral RNA (dsRNA) intermediates are formed, which are recognized and processed by the RNA interference machinery. The tick enzyme Dicer recognizes dsRNAs and cleaves these into vsiRNAs of predominantly 22 nucleotides (nt) [55,60]. In the current study, active USUV replication in artificially and naturally infected ticks was confirmed by the detection of USUV specific vsiRNAs. A clear peak of 22nt vsiRNAs was shown in pools obtained from both laboratory exposed ticks as well as from ticks collected from wild birds. These 22nt vsiRNAs in the tick pools had a stronger bias to the structural region of USUV compared to the non-structural region, as observed previously in *I*. *scapularis* ticks infected with Powassan virus [61]. The 18-32nt vsiRNA profiles were consistent with studies in ticks [55,61]. 24-32nt vsiRNAs were also sequenced, but lacked typical piwi-interacting RNA (piRNA) signature, as previously reported.

We showed that USUV crossed the midgut barrier as we detected virus in the legs in both artificially infected and naturally infected ticks. Moreover, the virus crossed the salivary gland barrier as we detected USUV RNA in a bloodmeal after feeding of USUV injected ticks. Although USUV crossed these infection barriers, which are important bottlenecks in the transmission of viruses by vectors [62], the virus persistence significantly reduced over time and after transstadial transmission from nymphal to adult ticks. Furthermore, USUV replicated more slowly in *I*. *ricinus* cells compared to TBEV, but was able to replicate to similar titres after 14 days of infection. This, in addition to the natural life cycle of ticks, indicates that the efficiency of USUV infection in ticks is limited by unknown mechanisms. Genetic differences between these viruses most likely caused the differences in replication dynamics [63]. Other studies have shown that mosquito-borne viruses can replicate in tick cell-lines but at the cost of attenuated growth in their vertebrate and mosquito hosts [64]. Nevertheless, in ticks collected from birds, detection of USUV in legs as well as detection of 22nt vsiRNAs show that USUV can replicate and disseminate in *Ixodes* ticks under natural conditions.

Screening of ticks feeding on wild birds resulted in the detection of USUV RNA in ticks from 48 out of 622 birds (7.7%). In 2018, a year of high circulation of USUV in the Netherlands, increased detection of USUV RNA-positive ticks preceded increased reports of wild bird mortality [54]. Hence, screening of ticks feeding on wild birds for USUV RNA may be valuable to complement other surveillance efforts in wildlife to improve detection and understanding of USUV circulation. While USUV was not detected in questing ticks, most of the ticks obtained through this surveillance effort were collected in spring or early summer, before the typical peak of USUV infection in birds. In addition, no questing ticks were collected in 2018, a year of high circulation of USUV [54]. Collection of questing ticks in late summer and at locations where USUV is known to circulate in birds might provide further insights into the role of *I*. *ricinus* on USUV transmission, also given the observed decrease in USUV infectivity after the initial infection with the virus in ticks.

In the three cases were USUV RNA-positive birds carried positive ticks, viral genomic analysis revealed that the virus detected in the ticks was identical or closely related to the virus detected in their bird host. This strongly suggests transmission between ticks and bird hosts, without providing insight into the directionality of transmission. The USUV genomes detected in these birds and ticks belonged to lineage Africa 3, the most frequently detected USUV lineage in blackbirds in the Netherlands between 2016 and 2018 [36]. Notably, the majority of the swabs from birds carrying USUV RNA-positive ticks tested negative for USUV RNA. This could indicate that (i) these birds already cleared the viral infection, (ii) these birds were infected with viral loads below detection levels or (iii) these birds were not infected and USUV-positive ticks had acquired the virus from a previous bloodmeal. To investigate the directionality of transmission, a potential approach could consist of experimentally exposing birds to USUV via infected *Ixodes* ticks and feeding of ticks on USUV infected birds.

USUV RNA was detected in *I*. *ricinus* and *I*. *frontalis* ticks feeding on birds. *Ixodes ricinus* is a generalist tick species feeding on a large variety of mammals and birds, while *I*. *frontalis* is an ornithophilic tick species [16,65]. The opportunistic feeding behaviour of *I*. *ricinus* could help transmission between avian and mammalian species. The role of mammals in the transmission of USUV has hardly been investigated. Interestingly, USUV has been isolated from small rodents in Senegal [66], an important host for *I*. *ricinus* larvae and nymphs [67].

Although we show that *Ixodes* ticks can become infected with USUV and may transmit the virus to a new host, their role in the transmission of USUV in the field remains unclear. Circulation of USUV is mostly detected in summer and early autumn [54,68]. Maintenance of the virus in overwintering mosquitoes may be the primary route for overwintering of mosquito-borne viruses, and USUV has been detected in overwintering *Culex torrentium* in Poland [69–72]. Nevertheless, WNV-infected questing ticks have been found in spring in Russia [22,73], suggesting overwintering of WNV in tick populations [22,62,74,75]. Even though we detected USUV in ticks feeding on birds, we did not detect USUV in our questing ticks collected in early summer. In conclusion, we showed that *I*. *ricinus* ticks can sustain and transmit USUV under experimental conditions and can be infected with USUV while feeding on a bird host. However, in comparison to mosquito-borne transmission, the role of ticks in the epidemiology of USUV is expected to be minor.

## Supporting information

S1 Fig Distribution of 24-30nt vsiRNAs of USUV infected *Ixodes* ticks across the USUV genome. Percentage of viral reads mapping to the sense genome are depicted in blue. Panel A and B represent two pools of artificial blood fed *I*. *ricinus* ticks. The viral reads mapping to the antisense genome are depicted in red. vsiRNA read counts were normalized against the total small RNA library (21 million).
(EPS)

S1 Table Overview of questing ticks that were screened for USUV RNA, per location of collection. None of the ticks were found positive for USUV.
(XLSX)

S2 Table Overview of birds and their carried ticks from which USUV genomes were generated. (XLSX)

S3 Table Incidence rate ratio (IRR) of viral titres compared to different incubation periods in USUV infected *Ixodes ricinus* ticks. (XLSX)
